# Students’ performance, attitude, and classroom observation data to assess the effect of problem-based learning approach supplemented by YouTube videos in Ugandan classroom

**DOI:** 10.1038/s41597-024-03206-2

**Published:** 2024-04-25

**Authors:** Nicholus Gumisirizah, Joseph Nzabahimana, Charles M. Muwonge

**Affiliations:** 1 African Center of Excellence for Innovative Teaching and Learning Mathematics and Science (ACEITLMS), University of Rwanda College of Education (URCE), Kayonza, P.O Box 55 Rwamagana, Rwanda; 2 Mbarara Science and Technology (MUST), Mbarara, Uganda

**Keywords:** Education, Applied physics

## Abstract

In response to global demands, Uganda’s Vision 2040 seeks to transform the country into a modern and prosperous nation by implementing Sustainable Development Goal (SDG) 4, focusing on equitable and quality education. The 21st-century workforce requires individuals who can effectively navigate complex workplace challenges. This dataset was gathered from Form-2 Ugandan secondary school students (aged 12 to 15) across 12 schools in the Sheema District. The dataset comprises three types of data: students’ performance in a physics topic (simple machines), their attitudes toward problem-solving and critical thinking when learning physics using Problem-Based Learning (PBL) supplemented by YouTube videos, and classroom observations documented with the reformed teaching observational protocol (RTOP). The intervention of teaching using PBL was executed in 2022, collecting data from 973 lower secondary school students. The intervention involved three approaches: one group (144 students) received PBL along with YouTube videos, another group of 482 students received PBL alone, and a third group (347 students) was taught using the traditional method. This data article explains the study’s data creation, collection, and analysis process. The dataset holds significance for secondary school teachers, policymakers, and researchers, offering insights into the impact of PBL with and without ICT resources on learning physics and students’ attitudes toward these learner-centered approaches.

## Background & Summary

Physics education in secondary schools plays a vital role in developing students’ social, physical, leadership, and problem-solving skills. Understanding physics concepts equips learners to know how things work, enabling them to apply this understanding to real-life situations^1^. The physics teaching is structured around activity-based^2^ chapters and topics, emphasizing hands-on experiences^3^ and practical applicability in everyday life. However, many students find physics challenging, necessitating an active teaching approach. Teaching in physics remains dynamic and interactive, with teachers adopting various strategies to engage students actively. Reciprocal teaching involves dialogues between the teacher and small student groups, while peer collaboration fosters cooperative work on class activities. Problem-Based Learning (PBL)^4–6^ is a student-centered approach that encourages group-based learning and teacher facilitation. It has been widely adopted in various educational fields, promoting problem-solving in learning environments. Implementing PBL follows a five-stage process:

### Finding a problem

The teacher prepares a task for students to investigate, stimulating problem-solving abilities.

### Organizing ideas on the problem

Learners investigate the problem, generate ideas, and receive probing questions from the facilitator to stimulate critical thinking.

### Group work

The teacher facilitates the distribution of learners into groups, each focusing on solving a particular problem related to the main task. Responsibilities are assigned within each group, promoting cooperation.

### Present findings

Learners present solutions to the problem and receive feedback from peers, consolidating their learning outcomes.

### Generalizing

Problem-solving leads to the development of skills essential for solving complex, real-world situations. These skills, including problem-solving, creativity, communication, cooperation, and innovation, prepare students to adapt to change and overcome 21st-century challenges.

Integrating YouTube videos as Information and Communication Technology (ICT) tools within a PBL approach offers a multifaceted strategy to enhance physics education^7,8^. High-quality videos aligned with curriculum objectives introduce real-world problems and cater to diverse learning styles. Interactive features and accessibility allow continuous learning, and educators can curate playlists to align with curriculum goals. The flipped classroom model^9^ combines videos with problem-solving discussions^10^, creating a dynamic learning environment that deepens students’ understanding of physics concepts and their practical applications.

Physics is a subject that holds a significant position in promoting scientific literacy, critical thinking, and essential life skills. However, conventional teaching methods often struggle to engage and empower students in the subject matter effectively. This inadequacy is a pressing concern, as it can hinder students from developing a strong foundation in physics, which is essential for their academic and practical pursuits. This study was critically important due to the existing challenges within physics education in Ugandan secondary schools. Incorporating innovative teaching approaches, such as PBL supplemented by YouTube videos, becomes pivotal in addressing these challenges. These methods can enhance students’ comprehension of physics and nurture vital skills like problem-solving, creativity, communication, cooperation, and innovation. These skills are indispensable for students to thrive in a rapidly evolving, knowledge-driven world.

Sharing the data generated through this study is equally significant. It is a valuable resource for educators, policymakers, curriculum designers, and researchers. By making this data accessible, the study contributes to the ongoing efforts to improve the quality and relevance of secondary education in Uganda. Educators can utilize this data to adopt innovative and effective teaching methods that align with the goals of the educational system, ultimately enhancing students’ performance and fostering lifelong learning. Policymakers and curriculum designers can use the insights derived from this data to conduct essential reviews and make informed decisions about teacher competence and the adoption of innovative teaching methodologies. Furthermore, researchers in similar fields can leverage this data to understand better the impact of PBL and the use of multimedia resources in education. This data identifies gaps and challenges and offers potential solutions and avenues for further research.

This data-sharing article presents insights into the effects of Problem-Based Learning (PBL) supplemented by YouTube videos on students’ comprehension of simple machines in physics within Ugandan lower secondary schools. The research collected data from 973 students, encompassing both public and private schools in the Sheema district of Uganda. Three primary types of data were collected: students’ performance data, attitude data, and classroom observation data.

Performance data was acquired through a Physics Learning Achievement Test (PLAT), involving students from various school types and teaching methods. Attitude data were collected via two surveys, one focusing on problem-solving ability (AAPS) and the other on critical thinking ability (CTMS) under PBL with YouTube videos. The Approaches to Problem-Solving Survey (AAPS) and the Critical Thinking Motivational Scale (CTMS) are measurement tools commonly used in the field of physics. The AAPS assesses various strategies individuals employ when solving problems, while the CTMS evaluates motivational factors influencing critical thinking abilities. The Reformed Teaching Observation Protocol (RTOP) assessed classroom practices and teaching methods.

The dataset, available in raw, filtered, and analyzed formats, offers valuable insights into the impact of innovative teaching methods on student performance, attitudes, and classroom practices. It addresses critical questions about the effectiveness of PBL approaches, with potential implications for science education in Uganda.

This dataset intends to assess the impact of PBL when supplemented with YouTube videos on Ugandan form-2 lower secondary schools in learning simple machines. The following are the research questions:

- To what extent do PBL and PBL supplemented with YouTube videos enhance students’ conceptual understanding of simple machines in physics?
- What are the problem-solving and critical thinking levels brought by learning with PBL supplemented by YouTube videos?
- How is physics teaching reformed when learning simple machines in physics with PBL supplemented by YouTube videos?
- Are there differences in students’ academic achievement for school type (government alongside private school)?

## Methods

### Ethics statements

The research project rigorously adhered to ethical standards established by the University of Rwanda College of Education’s (UR-CE) Research and Innovation Unit under the ethical protocol number Ref. 03/DRI-CE/078/EN/gi/2021, dated 30th November 2021. All necessary permissions were obtained systematically and ethically, as outlined in the research project description. Here is a summary of the ethical considerations and recruitment process:

#### Ethical protocol

The research project adhered to the ethical standards and principles of the UR-CE)‘s Research and Innovation Unit. The protocol number and approval date are explicitly mentioned, demonstrating a formal ethical review.

#### Permissions from authorities

The Ministry of Education and Sports obtained formal permission to access schools through the Permanent Secretary’s (PS) office. The PS communicated with the Chief Administrative Officer (CAO), District Education Officer (DEO), and Resident District Commissioner (RDC) to secure the necessary support for the study.

#### Engagement with schools

With the approval from the CAO, the DEO contacted school heads to inform them about the research study. The school heads responded positively and even provided physics teachers with three-day problem-based learning (PBL) training as part of the research. It is worth noting that all participating teachers held teaching qualifications, and as part of the research process, we provided them with a three-day training session specifically focused on implementing PBL interventions. This training aimed to ensure consistent delivery of PBL across treatment classrooms and schools, thereby mitigating variations attributable to individual teaching styles.

#### Informed consent

Teachers and students, with parental consent, willingly participated in the research study. Informed consent forms were signed, indicating they fully understood the study’s purpose, procedures, potential risks, and benefits. Anonymity was ensured for students by not including their names on the test papers.

#### Sampling

The research employed purposive sampling to select 973 students from 12 schools. These schools were divided into three groups, each with a different teaching method: PBL with YouTube videos, PBL alone, and Traditional teaching.

#### Geographic considerations

Schools were selected from different town councils at extreme ends of the district, sharing similar characteristics suitable for the study. This approach helps ensure that the study’s findings are robust and generalizable.

### Research design

The study utilized a non-equivalent comparison group pre/post-test design (Creswell, 2012). The study involves Form 2 students from six Sheema District, Western Uganda schools. Three selected schools were public, while the remaining three were private, offering a diverse representation of school types in the district. The selection of schools was purposeful, aiming to ensure diverse representation and maximize the study’s validity. This approach allowed for the strategic allocation of schools to treatment or control groups based on specific criteria pertinent to the research objectives. Notably, the selection criteria considered factors such as geographical location, school size, academic performance, and availability of resources to ensure a balanced representation of different educational contexts. The traditional method, characterized by conventional lectures supplemented with textbooks and teacher-centered content delivery, was employed in control group schools. Students in this group primarily learned through note-taking with minimal demonstrations. Conversely, four other secondary schools were designated as the first treatment group, where Problem-Based Learning (PBL) was implemented. Four additional schools comprised the second treatment group, which utilized PBL supplemented by educational YouTube videos. These groups collectively engaged in constructing knowledge and enhancing conceptual understanding. The participants in the study were form-2 students, ranging in age from 12 to 15 years, who were already enrolled in the schools.

We provide a performance (achievement) test to all 973 students before and after teaching interventions in all groups. We administered an attitude survey (motivation scale) and observed classes in the group that used PBL and YouTube videos. Table 1 presents the sample size under the teaching intervention of design groups implemented.

**Table 1:** Sample size and teaching intervention delivered.

Teaching intervention	Type of school	Research tool	Sample size
Traditional	Public (government)	Performance test	208
	Private		139
PBL alone	Public (government)		227
	Private		255
PBL with YouTube videos	Public (government)	Performance test	74
	Private	Attitude survey	70
		Classroom observation	

The objective of the performance test was to gauge students’ grasp of conceptual understanding acquired through the implementation of a problem-based learning approach following the completion of the topic on simple machines. The test, spanning 25 minutes, consisted of ten questions sourced from practice exercises on simple machines within form-two secondary learners’ physics textbooks. The National Curriculum Development Center and the Ministry of Education and Sports in Uganda approved these textbooks. The examination encompassed themes outlined in the approved lower secondary curriculum physics syllabus, covering concepts like the applications of simple machines, mechanical advantage, velocity ratio, and efficiency of machines. Specific topics included levers (covering classes and applications), pulley systems (encompassing types, applications, mechanical advantage, velocity ratio, and efficiency), inclined planes (including applications, mechanical advantage, velocity ratio, and efficiency), wheel and axle (exploring understanding, applications, and velocity ratio), gears (addressing simplification of work, applications, and velocity ratio), and methods of enhancing machine efficiency. The test was validated by four researchers from Mbarara University of Science and Technology (MUST) and the University of Rwanda College of Education (URCE). Test 1 was scored in MS Excel with “IF EXCACT” function, while Test 2 was manually marked, and results were entered in the same software.

Attitude surveys were all adopted from existing literature. Critical Thinking Motivational Scale (CTMS) was used as our Survey 1 and was adapted from Valenzuela *et al*.^11^, while Attitudes and Approaches to Problem-Solving Survey (AAPS) was used as our Survey 2 and was adapted from Singh and Mason^12^ and available at Physport (https://www.physport.org/assessments/assessment.cfm?A=AAPS). Problem-solving and critical thinking are integral to effective physics education. They deepen students’ understanding by connecting theoretical concepts to real-world situations^13,14^. These skills encourage active engagement and foster analytical abilities, allowing students to break down complex problems. Additionally, they promote creativity, help apply theory to practice, and cultivate logical reasoning. Problem-solving and critical thinking prepare students for future challenges in scientific and engineering fields, encourage collaboration, boost confidence, and instill a mindset for lifelong learning. Incorporating these skills into physics teaching enhances academic performance and equips students with valuable personal and professional growth tools. We adopted all 19 items from CTMS and only 31 items from AAPS to meet our research aim. Thus, the last two items (32 and 33) in AAPS were removed as they were not related to the content delivered in our study. All these surveys were rated on a Likert scale (from strongly disagree to strongly agree). Items 1–4 are related to expectancy, items 5-8 to attainment, items 9-12 to utility, items 13-16 to interest, and items 17-19 to cost.

Classroom observation data was collected with the famous standardized reformed teaching observation protocol (RTOP) from Pibun and Sawada^15^ and is available at Pysport (https://www.physport.org/assessments/assessment.cfm?A=RTOP). RTOP proved its validity and reliable results across the globe^16–19^ with its potential to reveal reformed teaching while implementing a new teaching method. It comprises 25 statements where each item is evaluated on a 5-scale. It is scored 0 when such practice was not found in a lesson and 4 when a certain practice was very well described or observed in a delivered lesson. During classroom observation, an observer sits in the classroom and observes what the teacher and student do. He/she may take notes on what is happening but wait until the class is over to rate these 25 items.

## Data Records

All data described in this descriptor are deposited in figshare (https://figshare.com/articles/dataset/RTOP_Data_for_the_implementation_of_Problem-based_learning_in_a_Physics_classroom_Uganda/23974902)^20^

To evaluate the impact of PBL teaching intervention on students’ performance and attitude toward learning physics, we gathered three data types (performance, attitude, and observation) presented in five datasets (two performance tests, two attitude surveys, and one classroom observation).

### Students’ performance data

The student performance data comprises two datasets or MS Excel files. The first file contains data for test one titled *“Performance data _ Test 1 (Multiple choice) _ 12102022 figshare.”* This file contains data from ten multiple-choice questions. The file contains three sheets. The first sheet shows test items (all ten questions), the second presents pretest answer choices, and the third presents post-test answer choices or results. Each results sheet shows the school code (column B), student code (column C), school type (column D), and treatment group (column E) as variables. From column “F” to column “O” we see student answer choices under each test question. From column “Q” to column “Z” we marked the test (one score for each correct question). Column “AB” shows the percent score. Row “3” shows the expected correct answer, while row “4” shows variables and the number of test items.

The second file contains data for test two titled *“Performance data _ Test 2 (Problem solving) _ 12102022 figshare.”* This file contains data from ten-word problem kinds of questions. The file contains three sheets. The first sheet shows test items (all ten questions), the second presents pretest scores, and the third presents post-test scores or results. Each results sheet shows the school code (column C), student code (column D), school type (column E), and treatment group (column F) as variables. From column “G” to column “P” we see student scores under each test question. Column “R” shows the total score, while column “S” shows percent score. Row “3” shows the assigned score when each question’s expected correct answer was provided. Row “4” shows variables and several test items.

### Students’ attitude data

The student attitude data comprises two datasets or MS Excel files. The first file contains data for the first survey titled *“Motivation data _ Survey 1 (Critical thinking ability) _ 12102022 figshare.”* This file contains data from 19 items of critical thinking ability survey. The file contains two sheets. The first sheet shows the pre-test results, while the second shows the post-test results. Each sheet shows the school code (column C), student code (column D), school type (column E), and treatment group (column F) as variables. From column “F” to column “O” we see student answer choices under each test question. From column “G” to column “Y” we see student answers or agreement (1: STRONGLY DISAGREE, 2: DISAGREE, 3,: NEUTRAL, 4: AGREE, AND 5: STRONGLY AGREE) to each item of the survey. Row “2” shows the survey title, while row “4” shows the variables and number of survey items.

The second file contains data for the second survey titled *“Attitude data _ Survey 2 (Problem solving ability) _ 12102022 figshare.”* This file contains data from 31 items related to problem-solving ability in learning physics. The file contains two sheets. The first sheet shows the pre-test results, while the second shows post-test results. Each sheet shows the school code (column C), student code (column D), school type (column E), and treatment group (column F) as variables. From column “G” to column “AK” we see student answers or agreement (1: STRONGLY DISAGREE, 2: DISAGREE, 3,: NEUTRAL, 4: AGREE, AND 5: STRONGLY AGREE) to each item of the survey. Row “2” shows the survey title, while row “4” shows the variables and number of survey items.

### Classroom observation data

The file for classroom observation data is titled *“Classroom observation data _ RTOP for video & pbl group _ 12102022 figshare”* and contains only one sheet. From column “B” to column “C” we see RTOP while the following columns (D-AA) present data. Row “10” shows school codes, while row “5” shows several observations and frequencies under each school supplied with PBL and YouTube videos teaching intervention. The data range from 0 (never occurred) to 4 (very descriptive).

## Technical Validation

### PLAT

Initially, we had 20 problem-solving questions, but evaluators rated 10 as valid, which were included in the final administration. We also initially had 15 multiple-choice questions, and evaluators rated 10 as appropriate and aligned with the study objectives. A pilot study was conducted with 90 students to evaluate the face validity and reliability of the questions. We assessed the reliability of these items using a split-half method and obtained a high reliability (r = 0.87) for multiple-choice items and a medium reliability (r = 0.68) measured by the Pearson product-moment correlation coefficient for problem-solving items. The split-half reliability assumes that the two halves of the test are equivalent in difficulty and content^21^.

### CTMS and AAPS

During our pilot phase, the internal consistency of CTMS, assessed using Cronbach’s alpha, was found to be high (0.793) for all 19 items, medium (0.428) for expectancy, (0.411) for attainment, (0.686) for utility, (0.574) for interest, and (0.594) for cost. The AAPS exhibited an internal consistency reliability of Cronbach’s alpha = 0.685. It is important to note that the AAPS contains nine items formulated negatively. Therefore, for a positive attitude, students were required to respond with ‘Disagree’ or ‘Strongly disagree’ to these items (1, 3, 5, 8, 11, 12, 16, 23, and 30). Consequently, the reliability of the 22 positively formulated items was 0.601, while that of the negatively formulated items was 0.480.

### RTOP

Before observing actual classes, we underwent a 2-hour training session and watched and coded a YouTube classroom video on physics. The inter-rater agreement between the first author and the assistant exceeded 80% on two occasions, indicating the reliability of the data.

### Scope and potential limitations

In our study, we recognized the significance of investigating potential bias in the results obtained from students in both private and public schools. To ensure the credibility and robustness of our findings, we conducted a comparative analysis to determine whether any notable disparities existed between these two groups. Our data collection process was comprehensive, encompassing a diverse range of schools, including both private and public institutions. This approach allowed us to capture a broad spectrum of socioeconomic backgrounds and educational settings. The study itself involved Form-2 students who were enrolled in schools situated in different town councils at opposite ends of the district. Despite their geographical diversity, these schools shared pertinent characteristics relevant to our research objectives. To facilitate our investigation, we categorized these schools into distinct treatment groups, comprising PBL alone and PBL with videos, along with a control group following traditional teaching methods. Importantly, we deliberately chose to maintain the existing class arrangements in these schools. Our commitment to preserving each school’s established class organization and cultural norms guided this decision.

However, it is essential to acknowledge the limitations inherent in the research design. One notable limitation is the observation of attitudes, which was limited to the student group exposed to the PBL with the video teaching method. This restriction may impact the generalizability of the findings, as attitudes toward learning may vary among students exposed to different instructional methods. Future research endeavors could consider incorporating measures to assess attitudes across all treatment groups to provide a more comprehensive understanding of the intervention’s effects.

The current data files do not contain information on individual teachers due to the scope and focus of the study. These variables could include educators’ teaching experience, pedagogical approach, content knowledge, and instructional effectiveness. Since we recognize the significance of teacher impact, we would consider incorporating such variables in future research projects to provide a more comprehensive analysis of instructional effectiveness and its associated factors.

Regarding the decision to maintain existing class arrangements in schools, particularly considering cultural norms, it is crucial to recognize its potential influence on the study outcomes. The intervention’s impact may have been influenced by preserving the existing class structures, including student composition and dynamics. For instance, certain class arrangements may foster greater collaboration and engagement, while others may present challenges in implementing collaborative learning approaches such as PBL. Therefore, future studies could explore the relationship between class arrangements and instructional effectiveness to provide insights into optimizing learning environments.

## Usage Notes

### Value of the data

- The data presented is valuable and beneficial to science education in Uganda as it elucidates the status of students’ content knowledge and their perceptions about learning simple machines with PBL approaches.
- Policymakers and curriculum designers have the opportunity to conduct essential reviews that highlight the competence of teachers. This process can pave the way for advocating innovative and relevant teaching methodologies, subsequently informing the identification of professional development requirements for educators.
- Researchers in similar fields can re-use these data to measure the effect of PBL intervention on student achievement, identify gaps, and predict possible remedies. Thus, data can be analyzed using various variables such as teaching intervention and school type.

## Data Availability

No custom code was used.
